# Deep CNN Sparse Coding for Real Time Inhaler Sounds Classification

**DOI:** 10.3390/s20082363

**Published:** 2020-04-21

**Authors:** Vaggelis Ntalianis, Nikos Dimitris Fakotakis, Stavros Nousias, Aris S. Lalos, Michael Birbas, Evangelia I. Zacharaki, Konstantinos Moustakas

**Affiliations:** 1 Department of Electrical & Computer Engineering, University of Patras, 26504 Patras, Greece; 2 Industrial Systems Institute, Athena Research Center, 26504 Patras, Greece

**Keywords:** deep sparse coding, convolutional neural networks, signal analysis, respiratory diseases, medication adherence

## Abstract

Effective management of chronic constrictive pulmonary conditions lies in proper and timely administration of medication. As a series of studies indicates, medication adherence can effectively be monitored by successfully identifying actions performed by patients during inhaler usage. This study focuses on the recognition of inhaler audio events during usage of pressurized metered dose inhalers (pMDI). Aiming at real-time performance, we investigate deep sparse coding techniques including convolutional filter pruning, scalar pruning and vector quantization, for different convolutional neural network (CNN) architectures. The recognition performance has been assessed on three healthy subjects following both within and across subjects modeling strategies. The selected CNN architecture classified drug actuation, inhalation and exhalation events, with 100%, 92.6% and 97.9% accuracy, respectively, when assessed in a leave-one-subject-out cross-validation setting. Moreover, sparse coding of the same architecture with an increasing compression rate from 1 to 7 resulted in only a small decrease in classification accuracy (from 95.7% to 94.5%), obtained by random (subject-agnostic) cross-validation. A more thorough assessment on a larger dataset, including recordings of subjects with multiple respiratory disease manifestations, is still required in order to better evaluate the method’s generalization ability and robustness.

## 1. Introduction

The respiratory system is a vital structure vulnerable to airborne infection and injury. Respiratory diseases are leading causes of death and disability across all ages in the world. Specifically, nearly 65 million people suffer from chronic obstructive pulmonary disease (COPD) and 3 million die from it each year. About 334 million people suffer from asthma, the most common chronic disease of childhood, affecting 14% of all children globally [1]. The effective management of chronic constrictive pulmonary conditions lies, mainly, in the proper and timely administration of medication. However, as recently reported [2], a large proportion of patients use their inhalers incorrectly. Studies have shown that possible technique errors can have an adverse impact on clinical outcome for users of inhaler medication [3,4]. Incorrect inhaler usage and poor adherence were found to be associated with high COPD assessment test scores [5], short durations of COPD, high durations of hospitalization and high numbers of exacerbations.

Several methods have been introduced to monitor a patient’s adherence to medication. As a series of studies indicate, effective medication adherence monitoring can be defined by successfully identifying actions performed by the patient during inhaler usage. Several inhaler types are available in the market, among which the pressurized metered dose inhalers and dry powder inhalers are the most common. In any case, the development of a smart inhaler setup, that allows better monitoring and direct feedback to the user independently of the drug type, is expected to lead to more efficient drug delivery, thereby becoming the main product used by patients.

The pMDI usage technique is characterized as successful, if a certain sequence of actions is followed [6]. Appropriate audio based monitoring could help patients synchronize their breath with drug activation and remind them to keep their breath after inhalation, for a sufficient amount of time. Several methodologies that engage electronic monitoring of medication adherence, have been introduced in the past two decades [7], aiming to alter patient behavioural patterns [8,9]. In the field of inhaler based health monitoring devices, a recent comprehensive review by Kikidis et al. [10] provides a comparative analysis of several research and commercial attempts in this direction. Cloud based self-management platforms and sensor networks constitute the next step towards effective medication adherence and self-management of respiratory conditions [11,12].

In all cases, it is crucial to successfully identify audio events related to medication adherence. In this direction, several approaches have been proposed in the literature, presenting mainly decision trees or other state of the art classifiers, applied on a series of extracted features. However, the aforementioned methodologies come with high computational cost, limiting the applicability of monitoring medication adherence to offline processing or online complex distributed cloud-based architectures, that are able to handle the need for resources. Therefore, the demand for computationally fast, yet highly accurate, classification techniques still remains.

Motivated by the aforementioned open issues, this study lies on the same track as several data-driven approaches [13,14,15], presenting a method that recognizes the respiration and drug delivery phases on acoustic signals derived from pMDI usage. The main focus of this work is the investigation of acceleration aspects, namely filter pruning, scalar pruning and vector quantization, applied on convolutional neural networks (CNNs). The adaptation of such strategies allows to reduce computational complexity and improve performance and energy efficiency. The CNNs are trained to differentiate four audio events, namely, drug actuation, inhalation, exhalation and other sounds. Five different CNN architectures are investigated and the classification accuracy is examined as a function of compression rate. More specifically, the benefits of this work can be summarized in the following points:

- The presented methods are applied directly on the time-domain avoiding computationally expensive feature extraction techniques.
- The overall classification accuracy for the proposed CNN architecture is high (95.7%–98%), for both within and across subjects cross-validation schemes.
- A compression rate by a factor of 7.0 can be achieved with accuracy dropping only by 1%.
- The investigated deep sparse coding oriented strategies (Implementation of this work and a part of the dataset used to validate it, is available online at: https://github.com/vntalianis/Deep-sparse-coding-for-real-time-sensing-of-medication-adherence), namely filter pruning and vector quantization, allow compliance with real-time requirements and open the path for adaptation of the inhaler device into Internet of Things (IoT).

The rest of the paper is organized as follows: Section 2 presents an extensive overview on relevant literature, Section 3 describes the CNN architectures and our methodology to enforce sparsity, Section 4 presents the experimental setup and the evaluation study and, finally, Section 5 provides future directions on the analysis of inhaler sounds.

## 2. Related Work

This section examines classical and data-driven approaches on classification of inhaler sounds. Early methodologies encompass electronic or mechanical meters integrated into the device, activated with the drug delivery button. Howard et al. [16] reported the existence of several such devices, able to record the time of each drug actuation, or the total number of them. The use of audio analysis came up later as a method, which can characterize the quality of inhaler usage, while, also, monitoring the timings of each audio event. The classical audio analysis involves transformation of the time-domain into a set of features, mainly, in the frequency domain, including Spectrogram, Mel-Frequency Ceptral Coefficients (MFCCs), Cepstrogram, Zero-Crossing Rate (ZCR), Power Spectral Density (PSD) and Continuous Wavelet Transform (CWT). Subsequently, audio-based evaluation employs the extracted features via classification approaches to locate and identify medication-related audio events.

Holmes et al. [17,18,19] designed decision trees in the scientific sub-field of blister detection and respiratory sound classification. This study includes detection of drug activation, breath detection and inhalation-exhalation differentiation and provides feedback, regarding to patient adherence. As a first step, the audio signal is segmented into frames of specific length, with overlaps. The mean power spectral density is calculated for defined frequencies and is used as a threshold to differentiate between blister and non-blister sounds. Also, the maximum normalized amplitude and the time duration are used to remove false positive sounds. The algorithm, then, examines the mean PSD, in specific frequency band, as the last threshold for blister sounds categorization. At the second stage, this algorithmic approach detects breath sounds. In this case, the audio signal is first filtered to remove high frequency components above a threshold, using a low-pass type I 6th order Chebyshev filter. Many window techniques exist for the design of Finite Impulse Response (FIR) and Infinite Impulse Response (IIR) Filters [20,21,22], such as Hamming, Hanning and Blackman for FIR Filter design, and Butterworth and Chebyshev for IIR Filters.

After signal segmentation, one set of 12 MFCCs is calculated for each frame, forming a short-time Cepstrogram of the signal. Ruinskiy et al. [23] perform singular value decomposition to capture the most important characteristics of breath sounds obtained from MFCC calculations. They set an adaptive threshold, according to the lowest singular vector in the inhaler recording, and mark the singular vectors above this threshold as potential breath events. For the last threshold, at this stage, the ZCR is extracted for each frame. Finally, the algorithms find the differentiation between inhalations and exhalations. The mean PSD of identified breaths is calculated for a determined frequency band and is used as a threshold for classification. Then, the standard deviation of the ZCR was found to be higher for inhalations in comparison to exhalations and a value is set, from empirical observations.

Taylor et al. [24,25] used the CWT to identify pMDI actuations, in order to quantitatively assess the inhaler technique, focusing only on the detection of inhaler actuation sounds. As a step forward data-driven approaches learn by example from features and distributions found in the data. Taylor et al. [26] compared Quadratic Discriminant Analysis (QDA) and Artificial Neural Network (ANN) based classifiers using MFCC, Linear Predictive Coding, ZCR and CWT features.

Nousias et al. [13,27] compared feature selection and classification strategies using Spectrogram, Cepstrogram and MFCC with supervised classifiers, such as Random Forest (RF), ADABoost and Support Vector Machines (SVMs), demonstrating high classification accuracy.

Pettas et al. [15] employed a deep learning based approach using the Spectrogram as a tool to develop a classifier of inhaler sounds. The Spectrogram is swept across the temporal dimension with a sliding window with length w=15 moving at a step size equal to a single window. The features of each sliding window are inserted into a recurrent neural network with long-short memory units (LSTM), demonstrating high performance in transitional states where mixture of classes appear.

Ntalianis et al. [14] employed five convolutional networks, applied directly in the time-domain, and showed that CNNs can automatically perform feature extraction and classification of audio samples with much lower computational complexity, at similar or higher classification accuracy than classical approaches. Each model uses a vector of n=4000 samples reshaped in a two-dimensional array (250×16), that is introduced in the deep CNN. Evaluation was limited to five-fold cross-validation in a subject-agnostic way, in which different samples from the same subject might be part of the training and test set, respectively.

This study aims to build upon previous CNN-based approaches for the identification of inhaler events, by investigating also acceleration strategies. CNNs have been established as a reliable state-of-the-art, data-driven approach for biosignal classification [28,29,30,31,32]. The adaptation of acceleration approaches, including filter pruning, scalar pruning and vector quantization, aims to lead to lower computational complexity and higher energy efficiency, facilitating IoT targeted implementations. In Section 4 we present the classification accuracy of the aforementioned studies, aiming to compare results of previous studies with our current approach.

## 3. Monitoring Medication Adherence through Deep Sparse Convolutional Coding

This section provides a comprehensive analysis of the deep architecture employed to perform the inhaler audio classification. Based on a main convolutional neural network architecture, five different variations are being investigated. Furthermore, compression and acceleration strategies, namely filter and scalar values pruning and vector quantization, are also being analyzed.

### 3.1. Convolutional Neural Network Architecture

**Figure 1:**
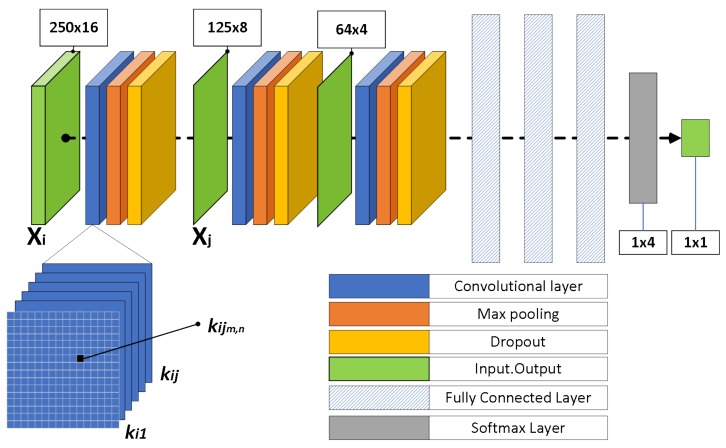
CNN Architecture.

The CNN architecture, presented in Figure 1, consists of three convolutional layers with a max-pooling layer, a dropout function [33] and four fully connected layers. Using this structure, five different CNNs were developed as presented in Table 1. For the convolutional kernels the stride is set equal to one with zero padding, in order to keep the shape of the output of each filter constant and equal to its input’s dimensionality. Every model utilizes the same sequence of layers, but with a different number of filters in the convolutional layers, or a different number of neurons in the fully connected layers, or different activation functions. Specifically, Table 1 presents the stacked layers for each model, the values of dropout layers, the number of filters in each convolutional layer, the number of neurons in fully connected layers and the activation function. In the fifth model, we select Exponential Linear Unit (ELU), due to the fact that the recordings contain both negative and positive values and ELU, in contrast to ReLu, does not zero out negative values. As far as the training parameters is concerned, the learning rate is set to 0.001, the batch size is equal to 100 and the categorical cross entropy loss function is employed. Training is executed through 5-fold cross validation with 20 epochs and Adam optimizer. In order to train the five CNN architectures, raw recordings in the time domain are directly used as input. The initial audio files contain multiple events, namely inhalation, exhalation, drug delivery and environmental noise. The final stage of preprocessing contains the formation of sound samples of 0.5 s duration (i.e., 4000 samples) collected with a sliding step of 500 samples. Only samples with unique classes are retained in the dataset. For a given convolutional layer, the previous layer’s feature maps are convolved with learnable kernels and passed through the activation function to form the output feature map described by Equation (1).

(1) $$x_{j}^{\ell }=f\left(\sum_{i\in M_{j}}\left(x_{i}^{\ell -1}*k_{ij}^{\ell }\right)+b_{j}^{\ell }\right),$$

where $M_{j}$ represents a selection of input feature maps. The output is fed to a set of four dense layers. The aforementioned architectures were chosen experimentally to keep the classification accuracy high and, simultaneously, the computational complexity as low as possible. We also experimented with both shallower and deeper architectures, but did not observe any further improvement.

For the implementation we used NumPy and SciPy, mainly for data mining and numerical computation tasks, as they are the fundamental packages to define, optimize and evaluate mathematical expressions, for scientific computing. These libraries also optimize the utilization of GPU and CPU, making the performance of data-intensive computation even faster. We developed this approach using Scikit-learn, which is built on top of the two aforementioned libraries and, also, using Tensorflow, which focuses on building a system of multi-layered nodes (multi-layered nodes system with high-level data structures). That allowed us to train and run the convolutional networks on either CPU or GPU. We, furthermore, used Pandas, which focuses on data manipulation and analysis (grouping, combining, filtering, etc.) and Keras, which is a high-level neural networks API, running on top of Tensorflow. Lastly, we used Matplotlib, as a standard Python library for data visualization (2D plots and graphs).

### 3.2. Filter Pruning

In order to reduce the computational requirements of the developed CNN architectures, we performed filter pruning as described in Reference [34], aiming to remove the less significant kernels in the convolutional layers, without deteriorating performance. In particular, we evaluate the contribution of each kernel, at the output of the layer, by calculating the sum of its absolute weights. For a convolutional layer with an input feature map of

(2) $$x_{i}\in {ℜ}^{h_{i}\times w_{i}\times n_{i}},$$

where $h_{i}$, $w_{i}$, $n_{i}$ are the height, the width and the number of channels of the input respectively, the output feature response has a shape of

(3) $$x_{i+1}\in {ℜ}^{h_{i+1}\times w_{i+1}\times j_{i+1}},$$

after applying $j_{i+1}$ filters with a kernel matrix $k\in {ℜ}^{k_{1}\times k_{2}\times n_{i}}$. The filter pruning process can be summarized in the following steps:

(1) Compute the sum $s_{j}=\sum_{l=1}^{n_{i}}\sum k$ of the absolute values of the weights in each filter.
(2) Sort $s_{j}$ and remove *n* filters with the lowest sum $s_{j}$.
(3) The rest of the weights remain unchanged.

For the filter pruning operation, two different approaches can be employed. Each layer can be pruned independently from others, referred to as independent pruning, or by ignoring the removed filters, also referred to as greedy pruning. The removal of a filter in the *i*-th layer leads to the removal of the corresponding feature map, which in turn leads to the removal of the kernels that belong to the i+1th layer and are applied on the aforementioned feature map. So, with independent pruning we sort the filters by taking into consideration the sum of the weights in these kernels. On the other hand, greedy pruning does not include them in the computation of the sum. Note that both approaches produce a weight matrix with the same dimensions and differ only on the filters chosen to be pruned. Additionally, in order to affect the accuracy of the prediction model as less as possible, two training strategies can be followed: (1) Prune once and retrain. This approach executes the pruning procedure first and only after all layers are processed the classifier is retrained so that its classification accuracy reaches its initial value. (2) Iterative pruning and retraining. In contrast to the first approach, with this method, when a layer is pruned, the rest of the network is immediately retrained, before the following layer is pruned. In this way, we let the weights of the model adjust to the changes occurred in previous layers, thus retaining its classification accuracy.

### 3.3. Pruning Scalar Values

The parameters $k_{k_{1},k_{2},n_{i}}$ of the different filters within each layer of a CNN are distributed in a range of values, with standard deviation σ. Weights very close to zero have an almost negligible contribution to a neuron’s activation. To this end, a threshold $\ell \in [\ell_{min},\ell_{max}]$ is defined so that

(4) $$k_{k_{1},k_{2},n_{i}}=0\;if\;\left|k_{k_{1},k_{2},n_{i}}\right|<\ell \cdot \sigma ,$$

$\ell_{min}$ and $\ell_{max}$ define the range of values during hyperparameter optimization and were set experimentally to $\ell_{min}=0.1$ and $\ell_{max}=1.0$. It is important to clarify that we employ the standard deviation in order to control the maximum number of parameters to be pruned. Finally, this method was evaluated by directly carrying out pruning on every layer of the classifier and by retraining the layers that follow the pruned one.

### 3.4. Vector Quantization

#### 3.4.1. Scalar Quantization

One way to decrease the number of parameters in a layer is to perform scalar quantization to them [35]. For example, for a fully connected layer with weight matrix $W\in {ℜ}^{m\times n}$, we can unfold the matrix so that $W\in {ℜ}^{1\times m\cdot n}$ and perform k-means clustering as described by the following formula:

(5) $$\min\sum_{i}^{m\cdot n}\sum_{j}^{N_{cl}}{∥w_{i}-c_{j}∥}_{2}^{2}.$$

The codebook can be extracted from the $N_{cl}$ cluster centers $c_{j}$ produced by the k-means algorithm. The initial parameters are then assigned with cluster indexes to map them to the closest center. Consequently, we can reconstruct the initial weight matrix *W* as:

(6) $${\hat{W}}_{ij}=c_{z}$$

where

(7) $$\min_{z}{∥W_{ij}-c_{z}∥}_{2}^{2}.$$

In respect to the convolutional layers, first, we have to decide on which dimension the k-means algorithm is going to be applied [36]. In the *i* + 1 convolutional layer, the weight matrix is a 4-dimensional tensor $W\in {ℜ}^{k_{1}\times k_{2}\times n_{i}\times n_{i+1}}$, where $n_{i}$ is the number of channels of the input feature map and $n_{i+1}$ the number of channels of the output feature map. It is preferable to perform k-means along the channels of the output feature maps in order to reduce the computational requirements by reusing pre-computed inner products.

#### 3.4.2. Product Quantization

The main concept of product quantization is to split the vector space into multiple sub-spaces and perform quantization in each subspace separately. In this way, we are able to better exploit the redundancy in each subspace [35]. In particular, let a weight matrix of a fully connected layer $W\in {ℜ}^{m\times n}$. We partition it column-wise so that:

(8) $$W=\left[W^{1},W^{2},\ldots ,W^{s}\right],$$

where $W^{i}\in {ℜ}^{m\times (n/s)}$. We apply k-means clustering in each submatrix $W^{i}$:

(9) $$\min\sum_{z}^{n/s}\sum_{j}^{N_{cl}}{∥w_{z}^{i}-c_{j}^{i}∥}_{2}^{2},$$

where $w_{z}^{i}$ represents the *z*-th column of the sub-matrix $W^{i}$ and $c_{j}^{i}$ the column of the sub-codebook $C_{i}\in {ℜ}^{m\times N_{cl}}$. The reconstruction process is performed based on the assigned cluster and the codebook for every sub-vector $w_{z}^{i}$. So, the reconstructed matrix is:

(10) $$\hat{W}=\left[{\hat{W}}^{1},{\hat{W}}^{2},...,{\hat{W}}^{s}\right],\;\mathrm{where}$$

(11) $${\hat{w}}_{j}^{i}=c_{j}^{i},\;\mathrm{where}\;\min_{j}{∥w_{z}^{i}-c_{j}^{i}∥}_{2}^{2}.$$

It is important to highlight, that product quantization can be performed to either the *x*-axis or the *y*-axis of *W*, but most importantly the partitioning parameter *s* must divide *n*.

Regarding the convolutional layers, following the same idea as in scalar quantization, we split the initial space vector into sub-spaces along the channel axis and we perform k-means at the same dimension. Let a convolutional layer have *n* channels. Then, its weight matrix has a shape of $W\in {ℜ}^{k\times k\times c\times n}$. Introducing the splitting parameter *s*, each sub-space has a shape of $W^{l}\in {ℜ}^{k_{1}\times k_{2}\times n_{i}\times \left(n_{i+1}/s\right)}$.

### 3.5. Compression Rate and Computational Complexity

The objectives of the aforementioned algorithms are to reduce the storage requirements as well as the computational complexity of the classifier. This section provides an insight of the computation of compression and theoretical speed up that pruning and quantization achieve. As mentioned above, pruning causes sparsity by forcing weights to become zero. Consequently, these weights are not stored in memory achieving a compression rate, described by the following expression. This equation is used to compute the extent of the compression for a model when filter pruning is applied as well.

(12) $$compression\;rate=\frac{number\;of\;parameters\;in\;not\;pruned\;model}{number\;of\;non\;zero\;parameters\;after\;pruning}$$

On the other hand, quantizing with *k*-means clustering results in a more complex expression for the compression rate, because it depends on the magnitude of both codebook and the index matrix. In particular, for scalar quantization in fully connected layer, the codebook contains $N_{cl}$ values and only $log_{2}N_{cl}$ bits are necessary to encode the centers. Thus, if 32-bit (single-precision) representation is used for the calculations, the total amount of bits required to store is $32\cdot k+m\cdot n\cdot log_{2}\left(N_{cl}\right)$ and the compression rate that this method achieves, per layer, is:

(13) $$\frac{32\times m\times n}{32\times \;k+m\times \;n\times log_{2}\left(N_{cl}\right)}.$$

However, the burden of memory requirements due to the codebook is considered negligible, comparing to the requirements of index matrix. Therefore the compression rate can be approximated with the simpler formula [35]:

(14) $$32/log_{2}N_{cl}$$

In case of the convolutional layers the k-means algorithm is performed on the channels axis. Therefore the number of the weights required to be stored is:

(15) $$\mathrm{new}\_\mathrm{weights}=k_{1}\times k_{2}\times n_{i}\times N_{cl},$$

instead of the initial, which is:

(16) $$\mathrm{old}\_\mathrm{weights}=k_{1}\times k_{2}\times n_{i}\times n_{i+1}$$

and the compression rate can be computed by the following formula:

(17) $$\frac{32\times \mathrm{old}\_\mathrm{weights}}{32\times \mathrm{new}\_\mathrm{weights}+ch\times log_{2}\left(N_{cl}\right)}.$$

Note that the index matrix contains only as many positions as the number of channels in the convolutional layer. This lies on the fact that by performing clustering along the channels axis, we produce filters with the same weight values, thus we only need to map the initial filters to the new ones. Finally, the compression rate that product quantization approach presented in Section 3.4.2 can achieve, per layer, in the fully connected layer, is calculated by:

(18) $$\frac{32\times m\times n}{32\times m\times N_{cl}\times s+n\times log_{2}\left(N_{cl}\times s\right)}.$$

For this method both the cluster indexes and the codebook for each sub-vector should be stored. Regarding the convolutional layers, performing the k-means algorithm with $N_{cl}$ clusters along the channels the compression rate is calculated by:

(19) $$\frac{32\times k_{1}\times k_{2}\times n_{i}\times n_{i+1}}{32\times k_{1}\times k_{2}\times n_{i}\times N_{cl}\times s+n_{i+1}\times log_{2}N_{cl}}.$$

Next, we present the gain in floating point operations required to perform the classification task, when the aforementioned techniques are employed. Among these methods, pruning scalar values and scalar quantization on fully connected layer do not offer any computational benefit. The zeroed out weights are scattered inside the weight matrix of each layer and, therefore, they do not form a structural pattern. For example, this method does not guarantee that all zeroed out weights belong to a certain kernel or to a certain neuron. On the other hand, with filter pruning, we remove whole filters, reducing efficiently the computational burden. In a convolutional layer the amount of necessary operations depends on the dimensionality of the input feature map and the number of the weights in the layer. The total number of floating point operations in a convolutional layer is:

(20) $$n_{i+1}\times n_{i}\times k_{1}\times k_{2}\times h_{i+1}\times w_{i+1},$$

where $n_{i}$ is the number of channels of the input feature map, *k* the dimensionality of the kernel, $n_{i+1}$ the channels of the output feature map and $h_{i+1},w_{i+1}$ the height and the width of the layer’s output, respectively. The product of $n_{i+1}\cdot n_{i}\cdot k_{1}\cdot k_{2}$ determines the amount of the weights that the layer contains.

The pruning of a single filter saves $n_{i}\cdot k_{1}\cdot k_{2}\cdot h_{i+1}\cdot w_{i+1}$ operations from the current layer and $n_{i+2}\cdot k_{1}\cdot k_{2}\cdot h_{i+2}\cdot w_{i+2}$ from the next layer. These additional operations can be avoided, because the certain kernels of the next convolutional layer are also removed. Specifically, the aforementioned kernels are applied on pruned feature maps.

Regarding the fully connected layer, the amount of floating point operations (flops) can be directly calculated from the dimensions of the weight matrix of a layer. For the weights matrix $W\in {ℜ}^{m\times n}$, the number of flops is calculated as m·n. The value of *m* corresponds to the dimension of the layer’s input and *n* to the dimension of its output, which is equal to the number of neurons in each layer. In order to reduce the number of flops in a fully connected layer, we should remove neurons, decreasing the dimensionality of the layer’s output.

Performing scalar quantization in convolutional layers with $N_{cl}$ clusters along the channel axis, we need to execute $N_{cl}\cdot n_{i}\cdot k_{1}\cdot k_{2}\cdot h_{i+1}\cdot w_{i+1}$ operations per layer which results in a ratio of:

(21) $$\frac{N_{cl}\times n_{i}\times k_{1}\times k_{2}\times h_{i+1}\times w_{i+1}}{n_{i+1}\times n_{i}\times k_{1}\times k_{2}\times h_{i+1}\times w_{i+1}}$$

and by introducing the splitting parameter *s* to perform product quantization, the previous ratio becomes:

(22) $$\frac{N_{cl}\times s\times n_{i}\times k_{1}\times k_{2}\times h_{i+1}\times w_{i+1}}{n_{i+1}\times n_{i}\times k_{1}\times k_{2}\times h_{i+1}\times w_{i+1}}.$$

Finally, performing product quantization on fully connected layers with $N_{cl}$ clusters and *s* sub-spaces, the ratio of the flops required after quantization, to the flops required before quantization, is calculated as

(23) $$\frac{m\times N_{cl}\times s}{m\times n}=\frac{N_{cl}\times s}{n}.$$

## 4. Experimental Procedure And Evaluation

### 4.1. Data Acquisition

Audio recordings from pMDI use were received, using a standard checklist of steps, recommended by National Institute of Health (NIH) guidelines, as it was essential to ensure that the actuation sounds were accurately recorded. The data were acquired from three subjects, between 28 and 34 years old, who all used the same inhaler device loaded with placebo canisters. The recordings were performed in an acoustically controlled indoor environment, free of ambient noise, at the University of Patras, to reflect possible use in real-life conditions and to ensure accurate data acquisition. The study supervisors were responsible for inhaler actuation sounds and respiratory sounds and followed a protocol, that defined all the essential steps of pMDI inhalation technique. Prior training of the participants, on this procedure, allowed to reduce the experimental variability and increase the consistency of action sequences. Each participant annotated in written form the onset and duration of each respiratory phase, during the whole experiment. Also, the annotation of the different actions was subsequently verified and completed by a trained researcher and based on visual inspection of the acquired temporal signal. In total, 360 audio files were recorded with a duration of twelve seconds each [13,14,15].

**Figure 2:**
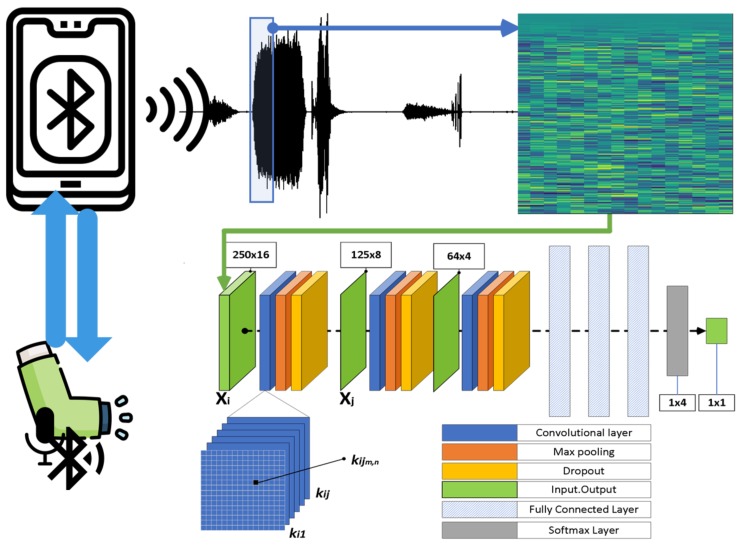
Overview of the processing pipeline.

The acoustics of inhaler use were recorded as mono WAV files, at a sampling rate of 8000 Hz. After quantization, the signal had a resolution (bit depth) of 16 bits/sample. Throughout the processing of the audio data, no further quantization on the data took place, except the quantization of the CNN weights into clusters of similar values of the convolutional and fully connected layer. The device for the recordings is presented in Reference [13]. Figure 2 depicts an overview of the processing pipeline. The sensor’s characteristics are 105 dB-SPL sensitivity and 20 Hz–20 kHz bandwidth. Each recording contains a full inhaler usage case. The first person (male) submitted 240 audio files, the second person (male) 70 audio files and the third subject (female) 50 audio files. Each subject, at first, breathes out and after bringing the device to their mouth he/she starts to inhale. Simultaneously, the subject presses the top of the inhaler to release the medication and continues to inhale until having taken a full breath. Then, breath holding follows for about 10 s and, finally, exhaling.

**Figure 3:**
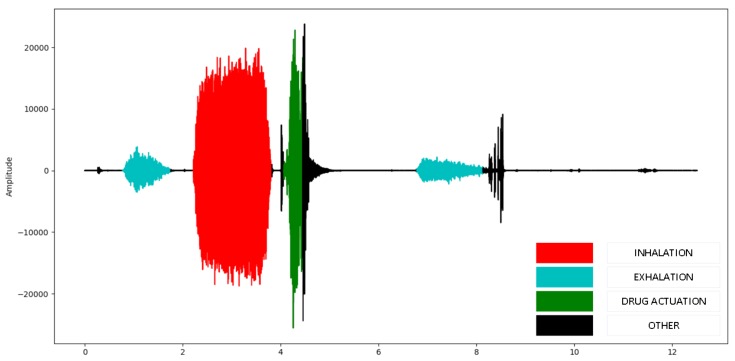
Annotated audio file of 12 s. Red color corresponds to inhalation, cyan to exhalation, green to drug activation and black to other sounds.

In order to train and test the proposed classifier, the audio recordings were segmented into inhaler activation, exhalation, inhalation, and noise (referring to environmental or other sounds) by a human expert using a graphical annotation tool [13]. A user interface visualizes the audio samples while the user selects parts of the audio files and assigns a class. The annotated part is stored in a separate audio file. A full audio recording timeseries example is presented in Figure 3, colored according to the annotated events. Any signal part that has not been annotated, was considered as noise, during the stage of validation. This dataset has the potential to allow in-depth analysis of patterns on sound classification and data analysis of inhaler use in clinical trial settings.

**Figure 4:**
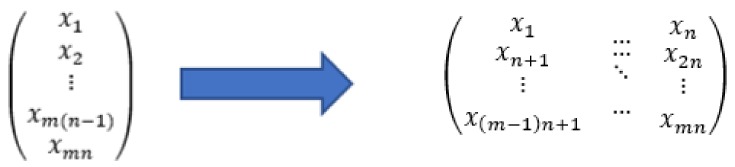
Illustration of reshaping of a vector into a two-dimensional matrix.

**Figure 5:**
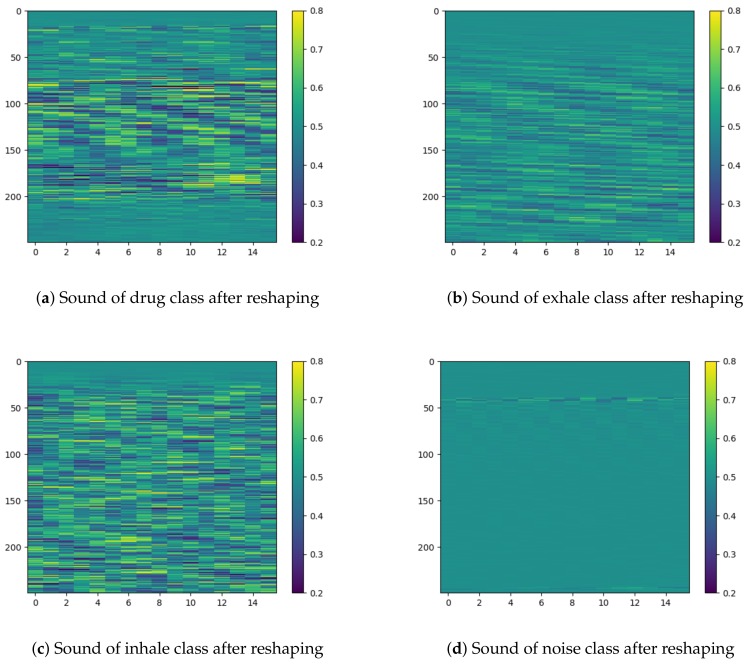
Visualization of the segmented audio files for each respiratory phase after the reshaping procedure.

Each sound sample has a total duration of 0.5 s, sampled with 8 kHz sampling rate and 16-bit depth. The audio files used for training and testing were loaded through appropriate libraries in a vector of 4000×1 dimension and, then, reshaping is performed in order to employ two-dimensional convolutions. In particular, the first 16 samples are placed in the first row of the matrix, the next 16 samples in the second, and so on, until a 250×16 matrix is constructed. An example of the reshaping procedure is given in Figure 4, while Figure 5 visualizes examples of sounds per class after this reshaping procedure.

### 4.2. Evaluation Schemes

The training and assessment of the five CNN models is performed in three different cross-validation settings. Firstly, we consider the *Multi Subject* modeling approach. In this case, the recordings of all three subjects are used to form a large dataset, which is divided in five equal parts used to perform five-fold cross-validation, thereby allowing different samples from the same subject to be used in training and test set, respectively. This validation scheme was followed in previous work [14] and thus performed, also, here for comparison purposes.

The second case includes the *Single Subject* setting, in which the performance of the classifier is validated through training and testing, within each subject’s recordings. Specifically, the recordings of each subject are split in five equal parts, to perform cross-validation. The accuracy is assessed for each subject separately and, then, the overall performance of the classifier is calculated by averaging the three individual results.

Finally, *Leave-One-Subject-Out (LOSO)* method is employed. With this approach we use the recordings of two subjects for training and the recordings of the third subject for testing. This procedure is completed, when all subjects have been used for testing, and the accuracy is averaged to obtain the overall performance of the classifiers.

### 4.3. Results

#### 4.3.1. Comparison with Relevant Previous Work

In order to better assess the contribution of the proposed approach, we first summarize in Table 2 the classification performance of previous state of the art algorithms that were presented in Section 2. In more details, Holmes et al. [17] presented, in 2012, a method that differentiates blister and non-blister events with an accuracy of 89.0%. A year later, Holmes et al. [18,19], also, developed an algorithm that recognizes blister events and breath events (with an accuracy of 92.1%) and separates inhalations from exhalations (with an accuracy of more than 90%). Later, Taylor et al. developed two main algorithms for blister detection [26,37] based on Quadratic Discriminant Analysis and ANN, and achieved an accuracy of 88.2% and 65.6%, respectively. Nousias et al. in Reference [13] presented a comparative study between Random Forest, ADABoost, Support Vector Machines and Gaussian Mixture Models, reaching the conclusion that RF and GMM yield a 97% to 98% classification accuracy on the examined dataset, when utilizing MFCC, Spectrogram and Cepstrogram features.

Pettas et. al [15] developed a recurrent neural network with long short term memory (LSTM), which was tested on the same dataset with this study and using the same modeling schemes, that is, *SingleSubj*, *MultiSubj* and *LOSO*. For the subject-specific modeling case the overall prediction accuracy was 94.75%, with higher accuracy in the prediction of breathing sounds (98%). Lower accuracy is demonstrated in drug administration and environmental sounds. Much higher accuracy is reported for *MultiSubj* modeling, where the training samples are obtained from all subjects and shuffled across time. It yielded a drug administration prediction accuracy of 93%, but a lower prediction accuracy of environmental sounds (79%), demonstrating a total of 92.76% accuracy over all cases. Furthermore, the *LOSO* validation demonstrated similar results, with the *SingleSubj* case. The high classification accuracy obtained by LSTM-based deep neural networks, is also in agreement with other studies [13,19]. Specifically, the recognition of breathing sounds is more accurate than the drug administration phase, which reaches a value of 88%, while the overall accuracy is 93.75%. In order to compare our approach with previous studies, we followed the same validation strategies for each different convolutional neural network architecture and summarize the comparative results in Table 3.

**Figure 6:**
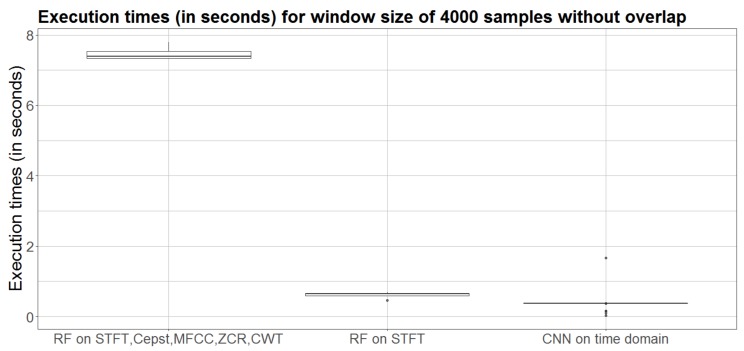
Comparison of the computational cost of our approach and other studies. Boxplots from left to right: RF with multiple features (mean time: 7.5 s), RF with only STFT (mean time: 0.6 s) and Model 5 of our CNN (mean time: 0.4 s).

From Table 2 and Table 3, it is apparent that the classification accuracy achieved by our approach does not exceed the performance of the relevant state of the art approaches. In fact, our approach performs, similarly, with the methods developed by Holmes et al. [17,18], Taylor et al. [24] and Pettas et al. [15], but the approach of Nousias et al. [13] outperforms our algorithm, mainly, for the drug and environmental noise classes. However, the utilization of a CNN architecture in the time domain allows for an implicit signal representation, that circumvents the need of additional feature extraction (e.g., in the spectral domain) and, thereby, results in significantly lower execution times. We compare the computational cost of Model 5 of our method with the Random Forest algorithm presented in Reference [13], both executed in the same machine (Intel(R) Core(TM) i5-5250U CPU @ 2.7 GHz). The results are summarized in Figure 6.

This figure highlights the gain in computational speed up of our approach, compared to the time consuming Random Forest algorithm with feature extraction. Specifically, Figure 6 shows that classification by RF, using multiple features, requires more than 7 s, whereas the CNN Model 5 requires less than half a second. Finally, it is important to note that our approach is faster even when only STFT is extracted and used as input to the Random Forest.

#### 4.3.2. Pruning Scalar Weights

In order to evaluate the performance of this algorithm, we present the classification accuracy as well as the compression rate, when no retraining is applied, in Table 4. The parameter *l*, which determines the threshold for pruning, varies from 0.1 to 1.0 with a step of 0.1. It is clear that when increasing the parameter *l* and consequently the threshold for pruning, the accuracy of the classifier decays. Among the five models, more robust to changes appears to be Models 1, 4 and 5, because they retain their performance above 90%, even when the parameter *l* is set to 0.8. On the other hand, model 3 and 4 show the worst performance dropping below 90% for intermediate values of *l*.

The results, presented in Table 5, corresponding to the approach that employs the retraining technique, show that the classifiers are able to adapt to the changes made in the previous layers, retaining their high accuracy, independently of the threshold defined by *l* and σ. It is worth mentioning that with this approach the lowest classification accuracy is 93% achieved by model 3, whereas model 5 reaches up to 96%, improving its initial performance. Additionally, we are able to compress the architectures two times more than the previous approach, where retraining process is not included. This occurs because retraining the network results in larger standard deviation of the weights in each layer, but with their mean value almost equal to zero. Thus, more weights will be zeroed out.

It should be highlighted that pruning scalar values can only reduce the memory requirements since there are fewer non zero weights. However, it does not perform structural pruning, meaning that it is uncertain if the pruned parameters belong to a particular filter or a neuron and therefore it does not improve the computational time.

#### 4.3.3. Pruning Filters in Convolutional Layers

In this section, we present the results for the evaluation of all five developed models, after applying the filter pruning method according to which the filters with the smallest magnitude are removed. We tested the classification accuracy of the pre-trained models for multiple combinations of pruned filters and, additionally, we investigated the effect of iterative pruning and retraining. For every model we chose to leave at least one filter at each convolutional layer. Thus, for Models 1, 2, 5 the number of the removed filters varies from 1 to 15, whereas for Models 3, 4 it is between 1 and 7. Table 6 and Table 7 present the performance of the models in terms of test loss and test accuracy, as well as results for the compression and the theoretical speed up of each architecture.

In Table 6 we observe that the classification accuracy of every model is significantly deteriorating, even at low compression rates. The reason for this is that filter pruning is employed on pre-trained models and therefore the values of their weights are not the optimal for the new, shallower architectures. In addition, Model 2 can be compressed at a larger scale than the others, due to its architecture. It has the most filters in the convolutional layers and, at the same time, the smallest number of neurons in the fully connected layers, as shown in Table 1, with the amount of parameters belonging to convolutional layers being approximately 19% of the total number of parameters, whereas for the other models it is 16% or lower. Note that even with half of the filters removed, the compression rate is low, indicating that the majority of the weights belongs to the fully connected layer. On the other hand, the removal of a filter reduces the computational requirements. For example, when we prune 2 out of 16 filters from model 1, the new, more shallow, architecture requires the 78% of the initial floating point operations to perform the classification task, providing a reduction of over 20%. Approaching the maximum number of the pruned filters (leaving only one filter), the required operations are, as expected, considerably reduced to only 1% of the operations required by un-pruned models.

A countermeasure against the drop of classification accuracy, due to filter removal, is the utilization of retraining technique, as described in Section 3.2. The results of filter pruning method with iterative retraining are shown in Table 7. It can be observed that the classification accuracy for all models except from Models 2, 4 remains over 90%, whereas for Models 2, 4 it drops to 35% and 87% when 15/16 and 7/8 filters are pruned in each layer, respectively. Thus, by applying this method we can significantly reduce the computational time without sacrificing efficiency. A characteristic example is Model 5, which reaches up to 95% classification accuracy, even with 13 filters pruned. For the same model, the respective performance achieved without retraining is 34%, while for both cases, the pruned models require 5% of the operations needed by the initial un-pruned architecture.

#### 4.3.4. Quantizing Only the Convolutional Layers

To evaluate the performance of the vector quantization method, we applied both scalar and product quantization to convolutional layers, as well as to fully connected layers of the network. This paragraph shows the classification accuracy of the developed models with respect to the compression rate and the number of required floating point operations, when the quantization methods are applied only on convolutional layers. As mentioned earlier, both scalar and product quantization are performed along the channel’s dimension. We tested different combinations regarding the number of clusters and the value of the splitting parameter *s*.

In particular, for scalar quantization the number of clusters varies between 1 and 8, whereas for product quantization we tried s=1,2,4 and the maximum number of clusters was set to 8, 4, 2 respectively. Note that for s=1 we essentially perform scalar quantization. Table 8 shows the classification accuracy and the achieved compression, as well as the speed up in terms of flops. It is clear, that by increasing the number of clusters and therefore the number of filters that contain different kernels, the accuracy of the classifiers increases as well. This originates from the fact that with more different filters more features of the input can be extracted in convolutional layers. It should be also mentioned that the compression rate achieved by this method, is lower than the rate achieved by filter pruning. This happens because an index matrix is required, to map the filters in the codebook to the filters in the original structure, which increases the memory requirements.

Concerning the amount of required operations in convolutional layers, as described before, it can be reduced with this approach by reusing pre-computed inner products. In particular, for similar convolutional kernels we only need to convolve one of them with the input feature map and then the result is shared. Then, the biases are added and the result passes the activation and pooling function, to produce the input feature map of the next layer. It is worth mentioning that the percentage of the required operations is directly proportional to the percentage of the filters needed to be stored. For example, clustering of 16 filters to 4 clusters causes a 25% reduction in required floating point operations. Again, comparing the flops for filter pruning and scalar quantization, the first is more efficient. This is because the removal of a filter reduces the dimensions of the next layer’s input feature map which is not the case for the scalar quantization.

Next, we evaluate the effect of product quantization on the performance of the models. Similarly, to scalar quantization on convolutional layer, we examine the fluctuation of the accuracy with respect to compression rate and the ratio of the required floating operations of the quantized architectures to the amount of flops for the initial structure, as shown in Table 9. The splitting parameter takes the values 1, 2, 4. As *s* increases, the number of clusters in each subspace decreases, since there are fewer filters. Because both the separation of the weight matrix of each layer and the k-means algorithm are performed on the channel axis, when s=1 the results are identical to those with scalar quantization. Additionally, the increase in the value of *s* result in a slight decrease in the classification accuracy of the model. For example, Model 1 with s=1 and clusters=4 reaches an accuracy of 93%, whereas with s=2 and clusters=2, a combination that produces 4 distinct filters in each layer, leads to 91%. This decrease indicates that apart from how many filters we group together, it is also crucial which filters are grouped. By splitting the original space in smaller sub-spaces, we narrow the available combinations of filters and, thus, filters that differ a lot from each other could be combined forming one cluster.

It is also important to note that the increase of the *s* parameter leads to a slight decrease of the compression rate. This is because with higher values of the splitting parameter, the lowest number of clusters in the weight matrix is increased as well. For example, for s=1 and clusters=4, the amount of different filters is 4, but if we set s=2 the respective amount would be 8, since we form 4 clusters in each subspace. Therefore, for the minimum number of clusters (1 cluster) and for s=1, one filter will be created. For s=2, two distinct filters will be formed and finally for s=4, four filters will have different weight values.

Concerning the performance of the architectures, with respect to the computational complexity, we observe in Table 9 that 75% of the initial flops can be avoided for Models 1, 2, 5 without any drop in classification accuracy. On the other hand, for the remaining models we save 50% of the initial required operations, with no drop in classification accuracy. For s=2, we are able to cut the majority for the operations with Models 1, 2, 5 reaching up to 94% accuracy with 38% of the initial amount of floating point operations. However, in order to achieve a classification accuracy higher than 90% we can reduce the amount of the operations by half at most. At 0.5 of the initial number of flops, model 3 reaches up to 95% and model 4 to 93%.

To sum up, quantizing convolutional layers using k-means algorithm, either with the scalar or the product method, we can compress the structure and at the same time we can speed up the production of the output feature map and, consequently, the prediction of the classifier. Between these two benefits, the computation gain is greater, since we efficiently can remove up to 75% of the operations required initially, whereas the maximum compression rate achieved reaches up to 1.2. This result is consistent with the theory suggesting that convolutional layers are computationally expensive and they do not add excessive memory requirements. Finally, for product quantization, increasing the value of the parameter *s*, the performance of the classifier is deteriorated.

#### 4.3.5. Quantizing Only Fully Connected Layer

Similarly, in this paragraph we present the results for scalar and product quantization, but in this case they are performed on the fully connected layers. For this approach we selected to perform quantization with k-means at the y axis of the weights matrix. In this way, we force the neurons to have the same output response and, therefore, we are able to reduce the computational requirements of the layers. Subsequently, we compare the requirements in storage and computation between convolutional and fully connected layer and validate that convolutions are time consuming, whereas fully connected layers significantly increase memory requirements. For Models 1, 2, 5 we perform scalar quantization with number of clusters up to 128 and for Models 3, 4 up to 52. We also executed tests for s=1,2,4 and clusters up to 32, 16, 8 respectively. It is important to mention that because we force some neurons to have the same output, we do not perform quantization at the output layer of the classifier i.e the last fully connected layer.

From the results shown in Table 10 it is clear that Models 1, 5, which share the same structure, have the same behaviour retaining their initial performance until a compression rate of 4.6. Furthermore, we are able to achieve a larger compression for Models 3, 4 because both of them have the shallowest convolutional structure, with three convolutional layers of 8 filters in each layer. This means that for these models the weights of fully connected layers occupy a greater portion of the total amount of the trainable parameters. It is important to highlight that we can compress model 4 more than six times and yet achieve a high classification accuracy up to 93%. Finally, for Models 2, 3 the maximum number of clusters is 52 because the last layer contains 16×4=64 weights and therefore there is no reason to increase it further. Also, as described above, scalar quantization does not contribute to the speed up of the classification task and this is why Table 10 does not contain the flops that the quantized models require.

The next approach to compress and accelerate the fully connected layers is product quantization through *k*-means algorithm. Table 11 and Table 12 present the classification accuracy, compression rate as well as the extent of the reduction of floating point operations for different number of clusters and different values of the splitting parameter *s*. In Table 11 Models 2, 3 we stop at 12 clusters, because they have a fully connected layer with 16 neurons and therefore there is no point in increasing the number of clusters beyond 12. Recall that quantization is performed on the columns of the weight matrix, that is, on the output response of a layer. It should be noted that the achieved compression rate is higher as the number of clusters is increasing than the respective rate with scalar quantization. This lies on the fact that the index matrix for this approach is smaller than the index matrix for scalar quantization, containing as many slots as the neurons in each layer are. Furthermore, the difference in the compression rate between the models is due to the difference between their structure. For example, Model 4 has smaller convolutional layers from Models 1, 2, 5 and larger fully connected layer from Model 3 resulting in a higher compression rate. Moreover, it is clear that by quantizing fully connected layers we do not have any gain in computational cost, since the smallest ratio with an acceptable performance is 0.977, which means that the quantized model needs to execute 97.7% of initial amount of floating point operations. Finally, Table 12 shows the performance of the classifiers for s=2 and 4. In this case, it should be highlighted that increasing the value of *s* the classification accuracy of the models decreases, despite the same compression rate. For example, model 1 achieves a classification accuracy of 94% with s=2 and 4 clusters but for s=4 and 2 clusters its accuracy drops to 90%.

#### 4.3.6. Combining Filter Pruning and Quantization

Finally, we investigate the combination of the aforementioned methods by applying filter pruning in the convolutional layers and quantization on fully connected layers. In this way, we are able to reduce the requirements of the classifier in both memory and computational power. The approach of the iterative training is selected for filter pruning, since it yields better results than the approach with no retraining. Firstly, we perform filter pruning in order to exploit the fact that the weights adjust to the changes and, then, quantization algorithm, either scalar or product, is executed. Below, we present the classification accuracy of the developed architecture, with respect to the amount of the pruned feature maps in convolutional layers and the number of clusters in fully connected layer.

**Figure 7:**
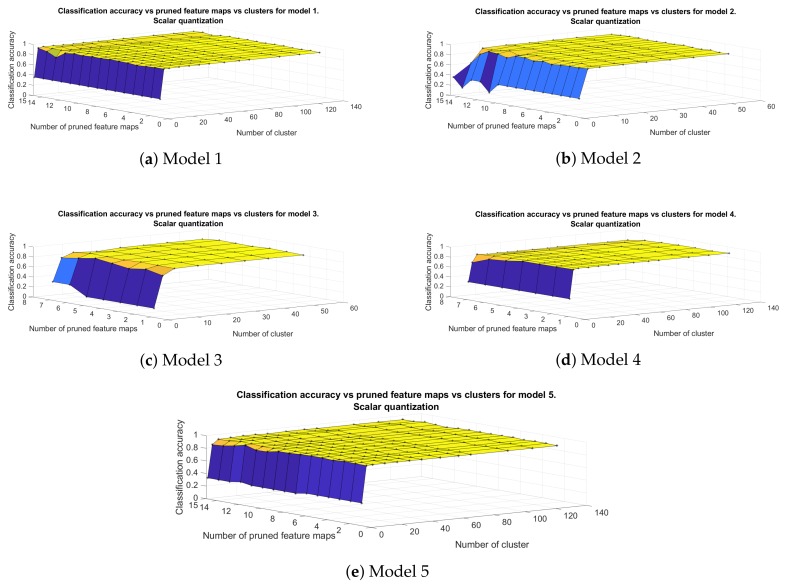
Classification accuracy of the different models in Table 1 that include filter pruning and scalar quantization. The horizontal axes represent the number of pruned feature map and number of clusters in fully connected layer, respectively.

Figure 7 shows how classification accuracy changes as the number of pruned feature maps or the clusters, produced with scalar quantization, increases. It is clear that the accuracy of all models, apart from model 2, depends mostly on the number of clusters in fully connected part of the classifier. When we perform k means on it, with clusters equal to 1, the classification accuracy drops to 35% (Models 1, 2, 4, 5) and 16% (Model 3). Model 2 reaches 91% or above when 14 out of 16 filters have been removed and for 8 clusters in each fully connected layer. However, when we prune 15 out of 16 filters its accuracy drops to 35% without improving when the number of clusters is increasing. On the other hand, the rest of the models keep their classification accuracy at high levels, even when their convolutional layers are left with only one filter. The best architectures seem to be Models 1 and 5, which achieve an accuracy over 90% with 8 clusters and even with 15 out of 16 filters removed.

**Figure 8:**
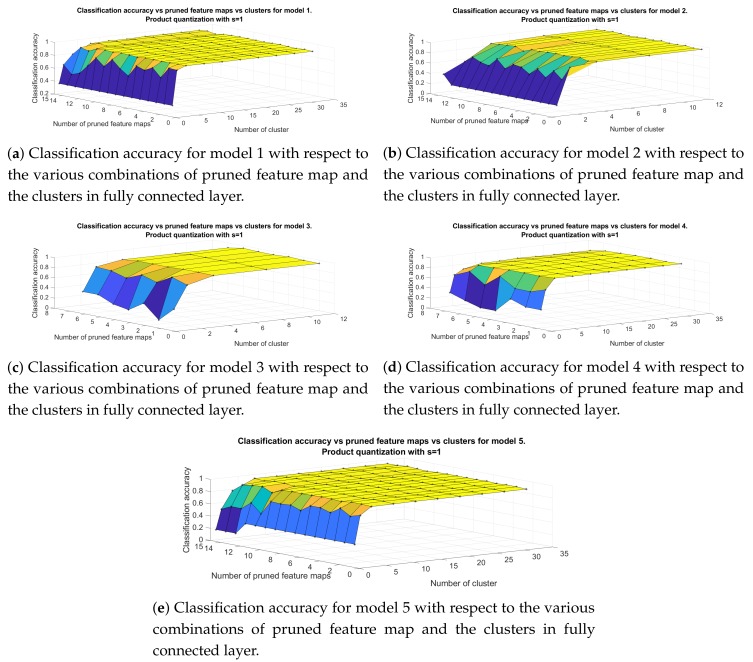
Classification accuracy for models in Table 1 for the approach that include filter pruning and product quantization with s=1.

Next, we proceed to the evaluation of combining filter pruning method with product quantization along y axis of the weight matrix of the fully connected layer. Figure 8 shows the classification accuracy of the developed models, versus the number of pruned feature maps and the number of clusters in each subspace, when the value of splitting parameter *s* is set equal to 1. For Models 1, 4, 5 the maximum amount of clusters is 32 whereas for Models 2, 3 is 12. Again, the parameter that affects mostly the performance of the classifier is the number of clusters produced by k-means algorithm. For cluster=1, hence when we force all neurons to have the same output response, the classification accuracy drops dramatically to 35% (Models 1, 5) and 16% (Models 2, 3, 4). It is also clear that the highest classification accuracy can be achieved with intermediate values of the parameters. For example, model 5 reaches up to 95.52% accuracy, which is the highest among our architectures, after pruning 7 feature maps and for 8 clusters in fully connected layer achieving 8 times compression of the initial structure. For the same level of compression model 1 achieves 94.66% (prunedfeaturemaps=7,clusters=8), model 2 93.97% (prunedfeaturemaps=7,clusters=8), model 3 reaches up to 93.8% (prunedfeaturemaps=2,clusters=4) and model 4 to 95.01% (prunedfeaturemaps=3,clusters=8).

**Figure 9:**
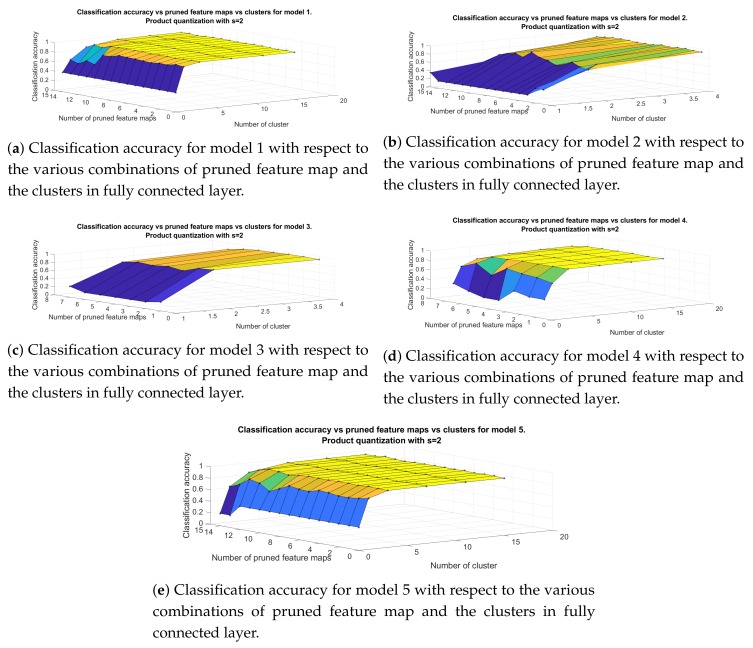
Classification accuracy for models in Table 1 for the approach that include filter pruning and product quantization with s=2.

Increasing the splitting parameter *s* to the value of 2 we take the results presented in Figure 9. Similarly to the results obtained from the previous experiments increasing the number of clusters in each subspace of the weight matrix we are able to improve the performance of the classifier. Again, the highest classification accuracy, 95.52%, is achieved by model 5 when we prune 3 filters from the convolutional layer and quantize the neural network at the back end, with 8 clusters in each subspace, leading to a compression factor of 3.5. However, we can compress it by a factor of 5 and at the same time achieve an accuracy up to 95.35%, which is an acceptable trade off between accuracy and compression, by quantizing it with 4 clusters instead of 8. For this compression rate model 1 yields 94.49% (prunedfeaturemaps=3,clusters=4), model 2 reaches up to 93.11% accuracy (prunedfeaturemaps=5,clusters=4), model 3 up to 92.6% (prunedfeaturemaps=1,clusters=4) and model 4 achieves an accuracy of 94.66% (prunedfeaturemaps=3,clusters=8). It is important to note that in order to achieve a compression rate of 8, we need, for model 5, to prune 7 filters and quantize with 4 clusters, but with a drop at classification accuracy of 2% achieving 93.97%. This result is consistent with those presented in previous sections, where it is shown that increasing the value of parameter *s*, the accuracy of the classifier at the same level of compression decreases.

**Figure 10:**
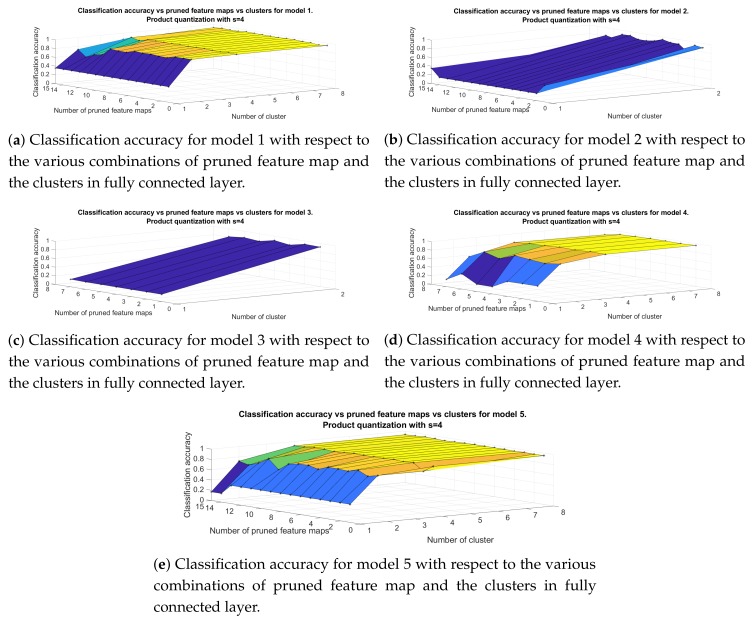
Classification accuracy for models in Table 1 for the approach that include filter pruning and product quantization with s=4.

Finally, Figure 10 presents the results for the classification accuracy with respect to the number of pruned feature maps and the number of clusters when the splitting parameter *s* is set to 4. For this approach the highest classification, 95.52%, is achieved by *Model 5* for the number of pruned filters set equal to five and for four clusters, which results in a compression rate of 4. The following table presents the number of clusters and pruned filters for each model, with a compression rate equal to 4.

Table 13 summarizes the results from the combination of filter pruning and scalar quantization in fully connected layers for the five proposed models and for the same compression rate. It can be seen that Models 1, 4, 5 yield similar classification accuracy despite the fact that Model 4 has fewer filters, while Models 2 and 3, which contain fewer parameters in the fully connected layer, fail to reach the same level of accuracy. In other words we can achieve high performance, even with limited number of filters, as long as we retain enough parameters at the fully connected layer. Overall, when comparing the compression techniques, Model 5 seems to achieve the best performance in most of the *MultiSubj* evaluation experiments. This fact, along with its superiority in *SingleSubj* and *LOSO* validation schemes, indicates that Model 5 is the most preferable among the five architectures.

## 5. Conclusions

Asthma adds an important socioeconomic burden both in terms of medication costs and disability adjusted life years. The accurate and timely assessment of asthma is the most significant factor towards preventive and efficient management of the disease. It outlines the need to examine the technological limitations for real time monitoring of pMDI usage, in order to create easy to use tools for safe and effective management. In this paper, we discussed on current medication adherence monitoring techniques, addressing related aspects that promote adherence with novel sensing capabilities. We, also, investigated acceleration approaches employing convolutional neural networks, trained to classify and identify respiration and medication adherence phases. Employing CNNs directly on the time domain, facilitated lower memory and processing power requirements. Evaluation studies demonstrate that the presented CNN-based approach results in faster execution time, requiring 0.4 s to perform a classification, whereas computationally expensive feature extraction approaches have a mean execution time of 7.5 s. Aiming at acceleration and compression we furthermore applied deep sparse coding strategies, namely filter pruning, scalar values pruning and vector quantization. Different CNN architectures were employed in order to assess the performance of deep sparse coding under various settings. The goal of such methodologies is to speed up the neural network outcome, allowing for real-time implementation, adaptable to IoT architectures and devices. Specifically, we achieve a compression rate of 6.0 in several cases, while maintaining a classification accuracy of 92%. The proposed work provides an experimental evaluation at a key area with renewed research interest, characterized by a high potential for novel improvements related to deep neural networks compression and acceleration. However, our approach is validated only through recordings of three healthy subjects resulting in a small dataset. More experiments with recordings from both healthy and subjects with respiratory disease should be carried out in order to thoroughly assess the presented approach and validate its potential in monitoring medication adherence.

## Figures and Tables

**Table 1:** Convolutional neural network (CNN) architecture variations for tested models.

Layers	Layer Parameters	Model 1	Model 2	Model 3	Model 4	Model 5
	**Filters**	16	16	8	8	16
**Convolutional Layer**	**Kernel Size**	4×4	4×4	4×4	4×4	4×4
	**Activation Function**	ReLu	ReLu	ReLu	ReLu	ELU
**Max Pooling**	**Kernel Size**	2×2	2×2	2×2	2×2	2×2
**Dropout**		0.2	0.2	0.2	0.2	0.2
	**Filters**	16	16	8	8	16
**Convolutional Layer**	**Kernel Size**	5×5	5×5	5×5	5×5	5×5
	**Activation Function**	ReLu	ReLu	ReLu	ReLu	ELU
**Max Pooling**	**Kernel Size**	2×2	2×2	2×2	2×2	2×2
**Dropout**		0.1	0.1	0.1	0.1	0.1
	**Filters**	16	16	8	8	16
**Convolutional Layer**	**Kernel Size**	6×6	6×6	6×6	6×6	6×6
	**Activation Function**	ReLu	ReLu	ReLu	ReLu	ELU
**Max Pooling**	**Kernel Size**	2×2	2×2	2×2	2×2	2×2
**Dense**	**Neurons**	64	64	64	64	64
	**Activation Function**	ReLu	ReLu	ReLu	ReLu	ELU
**Dense**	**Neurons**	128	32	32	128	128
	**Activation Function**	ReLu	ReLu	ReLu	ReLu	ELU
**Dense**	**Neurons**	64	16	16	64	64
	**Activation Function**	ReLu	ReLu	ReLu	ReLu	ELU
**Dense**	**Neurons**	4	4	4	4	4
	**Activation Function**	ReLu	ReLu	ReLu	ReLu	ELU
**Test Loss**		0.2413	0.2459	0.1891	0.2040	0.2145
**Test Accuracy**		0.9440	0.9397	0.9483	0.9586	0.9570

**Table 2:** State of the Art with multi-subject validation setting.

			Accuracy per Class (%)				Overall Accuracy (%)
			Drug	Inhale	Exhale	Noise	
Holmes et al. (2012)			89.0	-	-	-	89.0
Holmes et al. (2013-14)			92.1	91.7	93.7	-	92.5
Taylor et al. (2017)	QDA		88.2	-	-	-	88.2
	ANN		65.6	-	-	-	65.6
Nousias et al. (2018)	SVM	MFCC	97.5	97.7	96.1	96.7	97.0
		SPECT	97.5	94.9	58.9	95.4	86.6
		CEPST	99.4	98.6	98.2	98.8	98.7
	RF	MFCC	97.1	96.7	95.9	95.1	96.2
		SPECT	97.7	98.0	97.0	96.5	97.3
		CEPST	99.0	98.2	97.4	96.5	97.7
	ADA	MFCC	97.5	96.9	96.8	93.6	96.2
		SPECT	98.8	98.4	97.0	97.9	98.0
		CEPST	99.2	97.5	97.4	97.9	98.0
	GMM	MFCC	96.7	97.7	96.1	96.3	96.7
		SPECT	99.2	98.2	93.3	88.4	94.8
		CEPST	99.4	98.6	99.2	96.9	98.5
Proposed Approach	Model 1		88.4	99.4	92.2	85.7	94.4
	Model 2		83.9	99.1	97.5	81.9	94.0
	Model 3		86.4	98.9	94.5	80.2	94.8
	Model 4		83.6	98.7	96.2	83.4	95.9
	Model 5		86.7	97.9	98.3	85.5	95.7

**Table 3:** State of the Art with all validation settings.

			Accuracy per Class (%)				Overall Accuracy (%)
			Drug	Inhale	Exhale	Noise	
Pettas et al. (2019)	Single Subject		83.0	98.0	98.0	87.0	94.8
	Multi Subject		93.0	96.0	98.0	79.0	92.8
	LOSO		88.0	98.0	96.0	86.0	93.8
Proposed Approach	Model 1	Single Subject	71.5	99.3	98.1	93.1	97.4
		Multi Subject	88.4	99.4	92.2	85.7	94.4
		LOSO	100.0	96.3	98.8	-	93.2
	Model 2	Single Subject	76.7	99.7	96.6	80.9	97.6
		Multi Subject	83.9	99.1	97.5	81.9	94.0
		LOSO	100.0	93.6	95.4	-	83.4
	Model 3	Single Subject	65.3	99.6	98.9	84.2	97.5
		Multi Subject	86.4	98.9	94.5	80.2	94.8
		LOSO	100.0	92.2	89.0	-	98.0
	Model 4	Single Subject	68.4	99.6	99.0	84.9	98.2
		Multi Subject	83.6	98.7	96.2	83.4	95.9
		LOSO	85.7	82.6	99.2	-	86.0
	Model 5	Single Subject	85.0	99.5	99.5	95.0	98.0
		Multi Subject	86.7	97.9	98.3	85.5	95.7
		LOSO	100.0	92.6	97.9	-	96.2

**Table 4:** Evaluation of the performance for the developed architectures with the method of pruning scalar weights without retraining. Factor *l* corresponds to the percentage of the standard deviation used to determine the threshold for pruning.

	Factor *l*	0.1	0.2	0.3	0.4	0.5	0.6	0.7	0.8	0.9	1.0
Model 1	**Loss**	0.24	0.25	0.24	0.24	0.24	0.23	0.26	0.31	0.38	0.51
	**Accuracy (%)**	94.83	94.14	93.97	93.97	93.80	93.97	93.45	91.91	90.01	87.60
	**Compression Rate**	1.08	1.18	1.29	1.42	1.58	1.79	1.99	2.25	2.55	2.91
Model 2	**Loss**	0.24	0.26	0.28	0.33	0.60	0.69	1.54	0.98	0.97	1.34
	**Accuracy (%)**	93.97	93.45	93.28	91.56	84.16	82.09	58.86	65.95	69.53	55.93
	**Compression Rate**	1.08	1.19	1.31	1.45	1.63	1.84	2.10	2.40	2.77	3.19
Model 3	**Loss**	0.19	0.19	0.19	0.23	0.24	0.38	0.74	1.44	1.67	1.56
	**Accuracy (%)**	94.83	94.83	94.32	93.11	92.95	87.77	74.69	56.45	51.80	43.02
	**Compression Rate**	1.08	1.19	1.31	1.45	1.64	1.84	2.08	2.39	2.74	3.1712
Model 4	**Loss**	0.20	0.20	0.19	0.18	0.19	0.19	0.19	0.22	0.48	0.82
	**Accuracy (%)**	95.69	95.69	95.69	95.52	95.18	95.18	95.00	93.97	85.71	75.21
	**Compression Rate**	1.08	1.17	1.28	1.41	1.56	1.74	1.94	2.17	2.45	2.78
Model 5	**Loss**	0.21	0.21	0.21	0.20	0.20	0.21	**0.21**	0.39	0.31	0.64
	**Accuracy (%)**	95.70	95.87	95.87	95.87	95.53	95.01	**94.50**	88.83	90.20	79.89
	**Compression Rate**	1.08	1.17	1.29	1.42	1.57	1.76	**1.98**	2.24	2.54	2.89

**Table 5:** Evaluation of the performance for the developed architectures with the method of pruning scalar weights with retraining. Factor *l* corresponds to the portion of standard deviation used to determine the threshold for pruning.

	Factor *l*	0.1	0.2	0.3	0.4	0.5	0.6	0.7	0.8	0.9	1.0
Model 1	**Loss**	0.54	0.53	0.66	0.60	0.65	0.53	0.58	0.48	0.50	0.56
	**Accuracy (%)**	94.32	95.35	94.32	95.00	94.83	94.66	95.18	94.83	95.52	95.00
	**Compression Rate**	1.09	1.20	1.35	1.55	1.81	1.90	2.48	2.84	3.26	3.72
Model 2	**Loss**	0.51	0.57	0.53	0.52	0.48	0.64	0.61	0.54	0.50	0.51
	**Accuracy (%)**	93.80	94.32	94.14	94.66	94.49	94.32	94.66	95.00	95.18	94.49
	**Compression Rate**	1.09	1.22	1.38	1.62	1.74	2.35	2.81	3.28	3.74	4.28
Model 3	**Loss**	0.39	0.53	0.55	0.56	0.49	0.40	0.45	0.44	0.43	0.41
	**Accuracy (%)**	94.66	94.83	94.14	93.63	94.83	93.80	94.14	93.45	93.45	93.97
	**Compression Rate**	1.09	1.22	1.39	1.61	1.90	2.00	2.59	3.00	3.47	3.92
Model 4	**Loss**	0.44	0.52	0.49	0.60	0.43	0.52	0.50	0.50	0.47	0.50
	**Accuracy (%)**	95.52	95.18	95.35	95.18	94.83	94.83	95.00	95.00	95.18	94.83
	**Compression Rate**	1.09	1.20	1.35	1.55	1.63	2.11	2.44	2.82	3.21	3.63
Model 5	**Loss**	0.48	0.55	0.54	0.49	0.51	0.24	0.34	0.40	0.38	**0.39**
	**Accuracy (%)**	95.70	95.70	95.87	95.53	96.04	95.01	95.01	94.55	94.50	**94.50**
	**Compression Rate**	1.09	1.20	1.32	1.52	1.78	5.24	5.56	6.03	6.60	**7.14**

**Table 6:** Results for filter pruning with no retraining.

	Pruned Filters	1	2	3	4	5	6	7	8	9	10	11	12	13	14	15
Model 1	**Loss**	0.22	0.20	0.23	**0.24**	0.71	1.22	0.85	1.59	1.31	1.48	2.32	2.10	2.09	1.79	1.77
	**Accuracy (%)**	93.97	93.80	93.97	**93.97**	78.66	59.38	66.95	44.92	49.05	49.40	37.52	46.99	46.30	24.44	16.87
	**Compression Rate**	1.06	1.14	1.22	**1.31**	1.41	1.53	1.67	1.83	2.02	2.25	2.52	2.86	3.30	3.88	4.67
	**Flops**	0.89	0.78	0.68	**0.59**	0.50	0.42	0.35	0.28	0.22	0.17	0.12	0.09	0.06	0.03	0.01
Model 2	**Loss**	0.24	0.25	0.67	2.58	1.35	0.85	1.08	0.66	0.80	0.88	1.13	1.24	1.28	1.46	1.47
	**Accuracy (%)**	93.80	94.15	76.07	51.63	62.48	74.18	69.88	71.25	63.51	70.74	58.00	34.42	27.37	16.87	16.87
	**Compression Rate**	1.08	1.16	1.26	1.38	1.52	1.68	1.89	2.13	2.44	2.84	3.39	4.17	5.38	7.51	12.17
	**Flops**	0.89	0.78	0.68	0.59	0.50	0.42	0.35	0.28	0.22	0.17	0.12	0.08	0.05	0.03	0.01
Model 3	**Loss**	1.16	4.90	2.25	1.35	1.40	1.47	1.66	-	-	-	-	-	-	-	-
	**Accuracy (%)**	71.43	27.37	25.13	37.18	29.60	35.28	35.28	-	-	-	-	-	-	-	-
	**Compression Rate**	1.14	1.33	1.59	1.96	2.53	3.53	5.72	-	-	-	-	-	-	-	-
	**Flops**	0.79	0.61	0.44	0.31	0.19	0.10	0.04	-	-	-	-	-	-	-	-
Model 4	**Loss**	0.22	0.61	2.78	9.03	1.64	8.08	1.68	-	-	-	-	-	-	-	-
	**Accuracy (%)**	94.15	80.38	57.38	16.87	21.69	16.87	16.87	-	-	-	-	-	-	-	-
	**Compression Rate**	1.10	1.23	1.38	1.56	1.80	2.12	2.55	-	-	-	-	-	-	-	-
	**Flops**	0.79	0.61	0.45	0.31	0.20	0.11	0.05	-	-	-	-	-	-	-	-
Model 5	**Loss**	0.33	2.44	1.56	0.52	0.48	0.53	0.72	1.14	1.26	1.21	1.90	1.67	1.79	1.61	1.64
	**Accuracy (%)**	93.64	66.95	67.99	78.83	79.69	78.83	71.60	47.50	42.68	40.79	35.97	35.46	34.08	35.46	35.46
	**Compression Rate**	1.06	1.14	1.22	1.31	1.41	1.53	1.67	1.83	2.02	2.25	2.52	2.86	3.30	3.88	4.67
	**Flops**	0.89	0.78	0.68	0.59	0.50	0.42	0.35	0.28	0.22	0.17	0.13	0.09	0.0568	0.03	0.01

**Table 7:** Results for filter pruning with iterative retraining.

	Pruned Filters	1	2	3	4	5	6	7	8	9	10	11	12	13	14	15
Model 1	**Loss**	0.40	0.38	0.47	0.42	0.40	0.39	0.39	0.36	0.32	0.25	0.26	0.2704	0.29	0.20	0.25
	**Accuracy (%)**	94.32	95.18	94.32	94.49	95.18	95.18	94.32	94.66	95.01	94.84	94.84	93.63	92.08	94.32	92.94
	**Compression Rate**	1.06	1.14	1.22	1.31	1.41	1.53	1.67	1.83	2.02	2.25	2.52	2.86	3.30	3.88	4.67
	**Flops**	0.89	0.78	0.68	0.59	0.50	0.42	0.35	0.28	0.22	0.17	0.13	0.09	0.06	0.03	0.01
Model 2	**Loss**	0.40	0.50	0.37	0.36	0.34	0.25	0.32	0.30	0.29	0.36	0.27	0.21	0.21	0.26	1.32
	**Accuracy (%)**	93.97	93.46	95.18	93.80	94.15	95.52	94.15	94.66	93.97	93.46	94.66	94.66	93.97	93.11	35.46
	**Compression Rate**	1.08	1.16	1.26	1.38	1.52	1.68	1.88	2.12	2.43	2.84	3.39	4.17	5.38	7.51	12.17
	**Flops**	0.89	0.78	0.68	0.59	0.50	0.42	0.36	0.28	0.22	0.17	0.12	0.08	0.05	0.03	0.01
Model 3	**Loss**	0.33	0.32	0.25	0.26	0.20	0.18	0.27	-	-	-	-	-	-	-	-
	**Accuracy (%)**	93.63	94.15	94.84	93.29	93.80	94.15	91.05	-	-	-	-	-	-	-	-
	**Compression Rate**	1.14	1.33	1.59	1.96	2.53	3.53	5.72	-	-	-	-	-	-	-	-
	**Flops**	0.79	0.61	0.44	0.31	0.19	0.10	0.04	-	-	-	-	-	-	-	-
Model 4	**Loss**	0.37	0.33	0.36	0.25	0.28	0.23	0.35	-	-	-	-	-	-	-	-
	**Accuracy (%)**	95.87	94.49	95.15	94.84	93.80	92.94	87.78	-	-	-	-	-	-	-	-
	**Compression Rate**	1.10	1.23	1.38	1.56	1.80	2.12	2.55	-	-	-	-	-	-	-	-
	**Flops**	0.79	0.61	0.45	0.31	0.20	0.11	0.05	-	-	-	-	-	-	-	-
Model 5	**loss**	0.28	0.32	0.30	0.31	0.2854	0.28	0.23	0.32	0.29	0.31	0.26	0.2545	**0.25**	0.22	0.22
	**Accuracy (%)**	94.66	95.52	95.52	94.49	95.52	95.00	94.84	94.84	95.00	94.49	93.46	94.15	**95.18**	93.46	93.11
	**Compression Rate**	1.06	1.14	1.22	1.31	1.41	1.53	1.67	1.83	2.02	2.25	2.52	2.86	**3.30**	3.88	4.67
	**Flops**	0.89	0.78	0.68	0.59	0.50	0.42	0.35	0.28	0.22	0.17	0.13	0.09	**0.06**	0.03	0.01

**Table 8:** Results for scalar quantization on convolutional layers only.

	Number of Clusters	1	2	3	4	5	6	7	8
Model 1	**Loss**	4.61	0.38	0.33	0.26	0.25	0.23	0.24	0.24
	**Accuracy (%)**	16.87	90.36	92.94	94.32	94.84	94.84	94.84	94.66
	**Compression Rate**	1.18	1.16	1.15	1.14	1.12	1.11	1.10	1.09
	**Flops**	0.07	0.13	0.19	0.26	0.32	0.38	0.44	0.50
Model 2	**Loss**	4.35	0.28	0.25	0.22	0.23	0.23	0.24	0.25
	**Accuracy (%)**	16.87	92.08	93.80	94.32	94.32	94.32	94.66	94.32
	**Compression Rate**	1.18	1.17	1.15	1.13	1.12	1.10	1.10	1.08
	**Flops**	0.07	0.13	0.19	0.25	0.32	0.38	0.44	0.50
Model 3	**Loss**	10.57	0.40	0.25	0.20	0.20	0.18	0.19	0.19
	**Accuracy (%)**	16.87	89.16	93.80	94.49	94.84	95.01	94.84	94.84
	**Compression Rate**	1.10	1.08	1.07	1.05	1.04	1.03	1.01	1.00
	**Flops**	0.14	0.26	0.38	0.51	0.63	0.75	0.88	1.00
Model 4	**Loss**	10.88	1.08	0.24	0.22	0.22	0.22	0.21	0.20
	**Accuracy**	16.87	72.80	95.18	95.87	95.52	95.52	95.70	95.87
	**Compression Rate**	1.07	1.06	1.05	1.04	1.03	1.01	1.01	1.00
	**Flops**	0.14	0.26	0.39	0.50	0.63	0.75	0.88	1.00
Model 5	**Loss**	1.78	0.65	**0.24**	0.22	0.21	0.21	0.21	0.21
	**Accuracy (%)**	35.49	83.47	**94.49**	95.18	95.35	95.52	95.35	95.70
	**Compression Rate**	1.18	1.16	**1.15**	1.14	1.12	1.11	1.10	1.09
	**Flops**	0.07	0.13	**0.19**	0.26	0.32	0.38	0.44	0.50

**Table 9:** Product quantization for all combinations of *s* and clusters on convolutional layers only.

	Splitting Parameter	s = 1								s = 2				s = 4	
	**Clusters**	**1**	**2**	**3**	**4**	**5**	**6**	**7**	**8**	**1**	**2**	**3**	**4**	**1**	**2**
Model 1	**Loss**	4.61	0.35	0.33	0.26	0.23	0.23	0.24	0.24	2.89	0.35	0.28	0.27	2.65	**0.22**
	**Accuracy (%)**	16.87	91.74	92.94	93.80	94.66	95.18	94.66	94.66	16.87	91.91	94.15	93.46	39.76	**94.66**
	**compression rate**	1.18	1.16	1.15	1.14	1.12	1.11	1.10	1.09	1.16	1.14	1.11	1.09	1.14	**1.09**
	**Flops**	0.07	0.13	0.19	0.26	0.32	0.38	0.44	0.50	0.13	0.26	0.38	0.50	0.25	**0.50**
Model 2	**Loss**	4.35	0.27	0.25	0.22	0.22	0.23	0.23	0.24	1.69	0.28	0.23	0.24	1.24	0.26
	**Accuracy (%)**	16.87	91.91	93.63	94.32	94.49	94.49	94.66	94.66	37.00	92.94	93.97	94.32	53.87	93.11
	**Compression Rate**	1.21	1.20	1.18	1.16	1.15	1.13	1.12	1.10	1.20	1.16	1.13	1.10	1.16	1.10
	**Flops**	0.0690	0.13	0.19	0.25	0.32	0.38	0.44	0.50	0.13	0.25	0.38	0.50	0.25	0.50
Model 3	**Loss**	10.57	0.45	0.27	0.20	0.20	0.19	0.19	0.19	10.30	0.28	0.20	0.1892	2.21	0.19
	**Accuracy (%)**	16.87	45.13	27.00	20.17	19.91	18.84	18.62	18.92	17.04	95.01	95.01	94.83	38.21	94.84
	**Compression Rate**	1.10	1.08	1.07	1.05	1.04	1.03	1.01	1.00	1.08	1.05	1.03	1.00	1.05	1.00
	**Flops**	0.14	0.26	0.38	0.51	0.63	0.75	0.88	1.00	0.26	0.51	0.75	1.00	0.50	1.00
Model 4	**Loss**	10.88	1.00	0.22	0.22	0.23	0.22	0.21	0.20	11.93	0.28	0.25	0.20	4.41	0.20
	**Accuracy (%)**	16.87	74.35	95.70	96.04	95.52	95.52	95.70	95.87	16.87	93.46	95.35	95.87	37.52	95.87
	**Compression Rate**	1.07	1.06	1.05	1.04	1.03	1.02	1.01	1.00	1.06	1.04	1.02	1.00	1.04	1.00
	**Flops**	0.14	0.26	0.39	0.51	0.63	0.75	0.88	1.00	0.26	0.51	0.75	1.00	0.51	1.00
Model 5	**Loss**	1.78	0.65	**0.23**	0.21	0.21	0.21	0.21	0.21	2.01	0.64	**0.22**	0.21	1.69	0.29
	**Accuracy (%)**	35.59	83.99	**94.32**	95.01	95.70	95.35	95.70	96.04	35.46	83.47	**94.66**	95.35	47.85	91.91
	**Compression Rate**	1.18	1.16	**1.15**	1.14	1.12	1.11	1.10	1.09	1.16	1.14	**1.11**	1.09	1.14	1.09
	**Flops**	0.07	0.13	**0.19**	0.26	0.32	0.38	0.44	0.50	0.13	0.26	**0.38**	0.50	0.26	0.50

**Table 10:** Results for scalar quantization on fully connected layers only.

	Number of Clusters	1	4	8	16	24	32	40	52	64	72	96	112	128
Model 1	**Loss**	1.38	0.18	0.23	0.23	0.24	0.24	0.24	0.24	0.24	0.24	0.24	0.24	0.24
	**Accuracy (%)**	35.46	93.97	94.49	95.00	94.15	94.49	94.32	94.32	94.32	94.66	94.49	94.49	94.94
	**Compression Rate**	6.08	4.61	4.12	3.71	3.51	3.38	3.28	3.17	3.09	3.05	2.94	2.89	2.84
Model 2	**Loss**	1.38	0.44	0.18	0.26	0.25	0.26	0.27	0.26	-	-	-	-	-
	**Accuracy (%)**	16.87	86.75	94.66	93.97	93.80	93.63	93.46	93.63	-	-	-	-	-
	**Compression Rate**	5.26	4.15	3.76	3.43	3.26	3.15	3.07	2.98	-	-	-	-	-
Model 3	**Loss**	1.39	0.26	0.18	0.18	0.18	0.19	0.19	0.19	-	-	-	-	-
	**Accuracy (%)**	16.87	91.91	95.00	95.35	94.84	95.00	95.00	94.49	-	-	-	-	-
	**Compression Rate**	9.56	6.23	5.30	4.60	4.27	4.06	3.91	3.74	-	-	-	-	-
Model 4	**Loss**	0.35	0.23	**0.19**	0.20	0.21	0.20	0.20	0.20	0.20	0.20	0.20	0.20	0.20
	**Accuracy**	35.46	93.29	**95.52**	95.87	95.70	96.04	95.87	95.87	95.70	96.04	95.87	95.70	95.70
	**Compression Rate**	12.44	7.26	**5.10**	5.10	4.69	4.43	4.25	4.04	3.90	3.81	3.65	3.53	3.45
Model 5	**Loss**	1.37	0.19	0.21	0.21	0.21	0.21	0.21	0.21	0.21	0.21	0.21	0.21	0.21
	**Accuracy (%)**	35.46	94.32	95.70	95.52	95.00	95.87	95.52	95.70	95.87	95.70	95.87	95.87	95.70
	**Compression Rate**	6.08	4.61	4.12	3.71	3.51	3.38	3.28	3.17	3.09	3.05	2.94	2.89	2.84

**Table 11:** Product quantization for all combinations of clusters and s=1 on fully connected layer only.

	Splitting Parameter	s = 1									
	**Clusters**	**1**	**2**	**4**	**8**	**12**	**16**	**20**	**24**	**28**	**32**
Model 1	**Loss**	1.39	0.59	0.22	0.24	0.24	0.24	0.24	0.24	0.24	0.24
	**Accuracy (%)**	35.46	80.03	94.49	93.97	94.84	94.14	94.32	94.15	94.49	94.32
	**Compression Rate**	6.16	6.14	6.11	6.05	5.99	5.93	5.88	5.82	5.77	5.72
	**Flops**	0.99	0.99	0.99	0.99	0.99	0.99	0.99	0.99	0.99	0.99
Model 2	**Loss**	1.40	0.47	0.41	0.31	0.25	-	-	-	-	-
	**Accuracy**	16.87	83.30	88.12	92.25	93.80	-	-	-	-	-
	**Compression Rate**	5.29	5.27	5.25	5.20	5.16	-	-	-	-	-
	**Flops**	0.99	0.99	0.99	0.99	0.99	-	-	-	-	-
Model 3	**Loss**	1.40	0.76	0.19	0.19	0.20	-	-	-	-	-
	**Accuracy (%)**	35.46	63.85	94.32	95.18	94.66	-	-	-	-	-
	**Compression Rate**	9.74	9.69	9.59	9.41	9.24	-	-	-	-	-
	**Flops**	0.99	0.99	0.99	0.99	0.99	-	-	-	-	-
Model 4	**Loss**	1.37	0.57	**0.17**	0.20	0.20	0.20	0.20	0.20	0.20	0.20
	**Accuracy (%)**	35.97	84.68	**94.84**	95.69	95.52	95.69	95.52	95.52	95.87	95.87
	**Compression Rate**	13.12	13.03	**12.86**	12.57	12.31	12.05	11.81	11.59	11.37	11.15
	**Flops**	0.98	0.98	**0.98**	0.98	0.98	0.99	0.99	0.99	0.99	0.99
Model 5	**Loss**	1.36	0.41	0.21	0.20	0.21	0.21	0.21	0.21	0.21	0.21
	**Accuracy (%)**	35.46	88.81	95.35	95.52	95.87	95.87	96.04	96.04	95.70	95.70
	**Compression Rate**	6.16	6.14	6.11	6.05	5.99	5.93	5.88	5.82	5.77	5.72
	**Flops**	0.99	0.99	0.99	0.99	0.99	0.99	0.99	0.99	0.99	0.99

**Table 12:** Product quantization for all combinations of clusters and s=2,4 on fully connected layer only.

	Splitting Parameter	s = 2						s = 4			
	**Clusters**	**1**	**2**	**4**	**8**	**12**	**16**	**1**	**2**	**4**	**8**
Model 1	**Loss**	1.38	0.52	0.22	0.23	0.24	0.24	1.39	0.38	0.22	0.23
	**Accuracy (%)**	35.46	90.01	94.14	94.14	93.97	94.49	35.46	90.53	93.97	94.15
	**Compression Rate**	6.14	6.11	6.05	5.93	5.82	5.72	6.11	6.04	5.93	5.72
	**Flops**	0.99	0.99	0.99	0.99	0.99	0.99	0.99	0.99	0.99	0.99
Model 2	**Loss**	1.40	0.47	0.31	-	-	-	1.37	0.76	-	-
	**Accuracy**	16.87	81.41	91.05	-	-	-	16.87	76.25	-	-
	**Compression Rate**	5.27	5.25	5.20	-	-	-	5.25	5.20	-	-
	**Flops**	0.99	0.99	0.99	-	-	-	0.99	0.99	-	-
Model 3	**Loss**	1.43	0.64	0.95	-	-	-	1.48	0.37	-	-
	**Accuracy (%)**	16.87	79.69	94.84	-	-	-	16.87	90.71	-	-
	**Compression Rate**	9.69	9.59	9.41	-	-	-	9.59	9.41	-	-
	**Flops**	0.99	0.99	0.99	-	-	-	0.99	0.99	-	-
Model 4	**Loss**	1.37	0.57	**0.18**	0.20	0.20	0.20	1.38	0.46	**0.21**	0.20
	**Accuracy (%)**	37.00	85.54	**95.35**	95.70	95.52	95.70	36.60	88.12	**95.01**	95.52
	**Compression Rate**	13.03	12.86	**12.57**	12.06	11.59	11.15	12.86	12.57	**12.06**	11.15
	**Flops**	0.98	0.98	**0.98**	0.99	0.99	0.99	0.99	0.99	**0.99**	0.99
Model 5	**Loss**	1.36	0.35	0.19	0.21	0.21	0.21	1.37	0.35	0.21	0.20
	**Accuracy (%)**	35.46	91.74	95.18	95.87	95.52	96.04	35.46	89.84	94.66	95.70
	**Compression Rate**	6.14	6.11	6.05	5.93	5.82	5.72	6.11	6.05	5.93	5.72
	**Flops**	0.99	0.99	0.99	0.99	0.99	0.99	0.99	0.99	0.99	0.99

**Table 13:** Number of clusters and pruned filters for each model with a compression rate equal to 4.

Model	Accuracy	Pruned Feature Maps	Clusters	Compression Rate
**1**	95.01%	5	4	4
**2**	92.43%	2	2	4
**3**	91.74%	1	1	4
**4**	95.35%	5	4	4
**5**	**95.52%**	5	4	4

## Abbreviations

The following abbreviations are used in this manuscript:

CNN	Convolutional Neural Network
pMDI	pressurized Metered Dose Inhaler
MFCC	Mel-Frequency Ceptral Coefficients
RF	Random Forest
ZCR	Zero Cross Rate
